# Effects of the Active Choices Program on Self-Managed Physical Activity and Social Connectedness in Australian Defence Force Veterans: Protocol for a Cluster-Randomized Trial

**DOI:** 10.2196/21911

**Published:** 2021-02-24

**Authors:** Nicholas D Gilson, Zoe E Papinczak, Gregore Iven Mielke, Catherine Haslam, Jonas Fooken, Jim McKenna, Wendy J Brown

**Affiliations:** 1 School of Human Movement and Nutrition Sciences The University of Queensland Brisbane Australia; 2 School of Psychology The University of Queensland Brisbane Australia; 3 Centre for the Business and Economics of Health The University of Queensland Brisbane Australia; 4 Active Lifestyles Research Centre Leeds Beckett University Leeds United Kingdom

**Keywords:** military service veterans, self-managed physical activity, behavioral support program, psychological well-being, social connectedness, health service utilization, health service costs, physical activity, well-being, health professional, veterans, behavioral, support program

## Abstract

**Background:**

A stepped-down program is one in which clients transition from the care of a health professional to self-managed care. Very little is known about the effectiveness of stepped-down physical activity (PA) programs for military service veterans.

**Objective:**

This study will test Active Choices, a stepped-down behavioral support program designed to help Australian Defence Force veterans and their dependents who are clients of the Department of Veterans’ Affairs, transition from treatment by an exercise physiologist or physiotherapist to self-managed PA.

**Methods:**

The study is a parallel-group, randomized trial, with city-based exercise physiology or physiotherapy practices that recruit eligible Department of Veterans’ Affairs clients assigned to Active Choices or a comparison program. The study aims to recruit 52 participants (26 in each group). The Active Choices program will consist of 2 face-to-face (Weeks 1, 12) and 2 telephone (Weeks 4 and 8) consultations. During these sessions, the participant and Active Choices consultant will utilize an evidence-based resource booklet to review the key benefits of an active lifestyle, build an action plan for PA preferences, set and review goals, self-monitor progress relative to set goals, and discuss strategies to overcome PA barriers. Linking participants to local PA communities to overcome social isolation will be a program priority. The comparison program will consist of 2 consultations (Weeks 1 and 12) and use fewer behavioral support strategies (education, self-monitoring, and action planning only) than Active Choices. Outcome measures will be administered at baseline, end-intervention (12 weeks), and follow-up (24 weeks) to assess changes in moderate intensity self-managed PA, psychological well-being, and social connectedness. We will also measure health service utilization and costs as well as PA choices across the intervention period. End-intervention interviews will capture participant experiences.

**Results:**

Due to the impacts of the COVID-19 pandemic on human research activities in Australia, participant recruitment will commence when it is safe and feasible to do so.

**Conclusions:**

Findings will provide valuable pilot data to support up-scaling of the program and larger effectiveness trials with regional and rural as well as city-based Australian Defence Force veterans and their dependents.

**Trial Registration:**

Australian and New Zealand Clinical Trials Registry (ANZCTR): ACTRN12620000559910; https://www.anzctr.org.au/ACTRN12620000559910.aspx

**International Registered Report Identifier (IRRID):**

PRR1-10.2196/21911

## Introduction

The numerous health and psychosocial benefits that accrue through regular participation in physical activity (PA) are well established [1]. PA is particularly beneficial for older people (≥65 years) and plays an important role in retaining independence and life quality through promoting functional fitness, cognitive function, psychological health and well-being, and social connectivity [2].

While active aging can help maintain holistic health and prevent disease, general practitioners are increasingly referring older patients to PA specialists for the treatment and management of existing health conditions, such as high blood pressure, diabetes, or musculoskeletal issues [3]. In Australia, for example, where referrals to exercise physiologists (EP) and physiotherapists may be government-funded, there has been significant growth in the provision of allied health services to older people. Data from the Department of Veterans’ Affairs (DVA), the Australian Government department that provides support to Australian Defence Force (ADF) veterans and their dependents, many of whom are older adults, indicate that from the period of 2011/2012 to 2016/2017, there was a 51% increase in the number of funded services accessed by DVA clients. This was despite a 19% reduction in the number of DVA clients who were eligible to access allied health services [4]. Service uptake was significantly underpinned by large increases in the number of DVA clients who saw an EP or physiotherapist for treatments involving PA.

The benefits of referral to a PA specialist include receipt of expert care and tailored PA guidance during treatment. However, it has been argued that PA referral programs lack behavior change components that promote longer-term adherence [5]. Consequently, such programs do not enable or create channels for patients to “step-down” to self-managed PA after a course of allied health treatment. A stepped-down program is one in which patients transition from allied health care to self-managed behavior [6]. Thus, individuals take responsibility for initiating and maintaining their own PA regimes, instead of being dependent on supervision from a PA specialist.

Given the significant range of health disparities between military service veterans and the general population, stepped-down programs that promote self-management offer a means to support ongoing engagement in PA [7]. It is also the case that self-management is challenging and greater support for sustainability is obtained through engagement in PA with others [8]. This may have the added benefit of helping veterans deal with challenges of social disconnection that can come from losing long-standing connections to peers and support networks that enable positive health behaviors [9]. Engaging veterans in health promotion programs that emphasize social connectivity through PA may be an effective strategy not only for sustainability of PA but also for re-establishing social support networks that can help counter feelings of isolation and ill-health [10].

Recognizing the need to develop, implement, and evaluate scalable interventions that can empower healthy lifestyle choices and social connectivity for military service veterans, the aim of this study is to test “Active Choices,” a stepped-down behavioral support program for ADF veterans and their dependents. The 12-week program seeks to connect and engage participants with their local active communities as they transition from DVA-supported treatment by an EP or physiotherapist to self-managed PA. This proof-of-concept study will determine the impacts of the Active Choices program on clients’ moderate intensity self-managed PA, psychological well-being, and social connectedness and explore the potential effects on health service utilization and costs. The acceptability and feasibility of the program will also be examined.

## Methods

### Study Design and Setting

The study is a 24-week, parallel-group, randomized trial for DVA clients (ADF veterans and their dependents) who have been cleared by their treating EP or physiotherapist as able to safely transition to self-managed PA. EP or physiotherapist practices that we engage to recruit eligible participants will be randomly assigned to the Active Choices or comparison program, using an allocation ratio of 1:1.

Randomization will be performed using a simple randomization procedure, based on a computer-generated sequence. Allocation of practices will be concealed at the time of assignment, and members of the research team who will analyze the study data will be blinded to allocation. However, blinding of participants and those who will administer the interventions and outcome assessments will not be possible due to the nature of the program.

**Figure 1:**
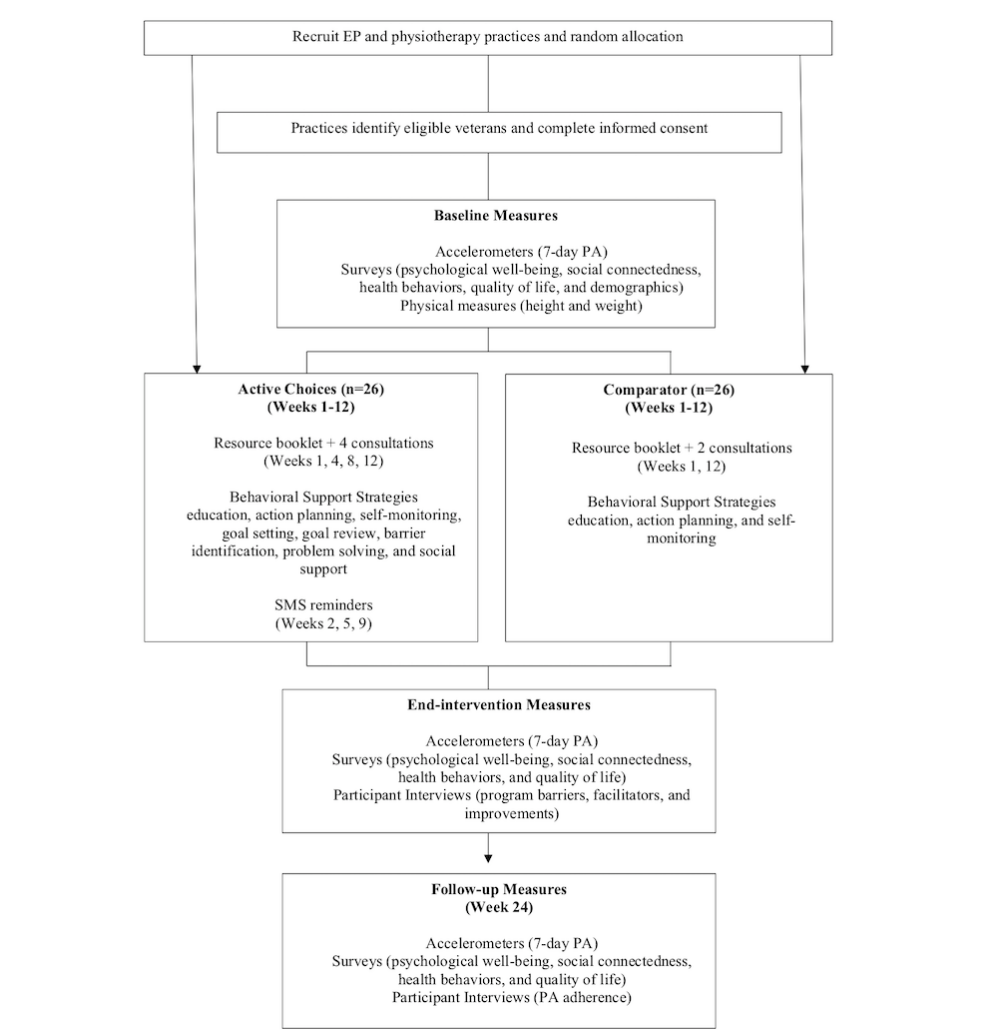
Participant flow through the trial. EP: exercise physiology; PA: physical activity.

Data will be collected from participants at 3 timepoints: baseline, end-intervention (Week 12), and follow-up (Week 24). The trial will be conducted in Brisbane, Australia. Intervention delivery and data collection will take place at the allied health practice the participant attends. A flow chart of the trial is shown in Figure 1.

### Sample Size

The reference parameters used to calculate the sample size were based on a recent large-scale survey conducted by our team, which examined accelerometer-measured PA in nearly 700 adults living in Brisbane, Australia. This study found participants accumulated an average of 10 minutes/day (70 minutes/week) of at least moderate intensity PA [11]. For our study, sample size was calculated based on change in accelerometer-measured PA, from baseline to 24 weeks, between the 2 groups, in order to detect a minimum average increase of 11 minutes/day (77 minutes/week) of moderate intensity PA. In addition to the PA observed in our reference study [11], this is equivalent to the minimum amount required to achieve standard Australian PA recommendations of 150 minutes/week of moderate intensity PA [12]. Assuming an average of 10 minutes/day (standard deviation of 12.7 minutes/day) at baseline, power of 80%, and significance level of 5%, our power analysis determined that a minimum sample size of 42 (21 in each group) is needed to detect a minimum average increase of 11 minutes/day in moderate intensity PA. To allow for 20% loss to follow-up and 10% noncompliance with PA device use, 52 participants will be recruited to the study (26 in each group).

### Recruitment and Eligibility Criteria

Following ethics approval, we will contact EP and physiotherapy practices in the Greater Brisbane region through Federal Government (DVA) and professional organization (Exercise and Sports Science Australia and Australian Physiotherapy Association) communication networks (eg, newsletters and social media) to inform them of the trial. EPs and physiotherapists interested in offering the program to their clients will act as “gatekeepers” for study recruitment. They will apply study inclusion criteria to DVA-funded clients and link eligible and interested clients with the study team for provision of informed consent. Eligibility criteria will be assessed a second time when obtaining informed consent from participants.

To be included in the study, participants must be ADF veterans and their dependents who are eligible to receive DVA-funded allied health treatment from an EP or physiotherapist, ≥50 years of age (this is based on recent data showing that 80% of the DVA treatment population are over 50 years old, with an average age of 67.25 years) [4], and identified by their treating EP or physiotherapist as able to safely transition from supervised treatment to self-managed PA. The DVA client population includes both ADF veterans and other eligible recipients of DVA-funded healthcare, mostly widow/ers of ADF veterans whose death was determined to be related to their military service.

Participants will be excluded if they are (1) under medical management for complex or chronic conditions that require supervised treatment from a health professional (inclusive of spinal cord injury, brain injury, severe mental health problems, chronic pain, stroke, amputations, and complicated orthopedic injury), (2) currently participating in another DVA-funded PA program, and (3) current serving ADF personnel.

### Interventions

#### Active Choices Program

Participants attending EP or physiotherapy practices allocated to the Active Choices intervention group will receive a 12-week program consisting of two 1-hour face-to-face consultations (held in Weeks 1 and 12) and two 30-minute telephone consultations (held in Weeks 4 and 8), which participants will complete individually with an Active Choices consultant.

The program is based on the COM-B Framework [13] and incorporates evidence-based behavior change strategies to support participants’ transition to self-managed PA. These strategies comprise education, goal setting, goal review, self-monitoring, social support, action planning, barrier identification, and problem solving. Strategy selection was also informed by the findings of a systematic review completed by the research team, which identified those utilized in previous intervention studies associated with increased self-managed PA among military service veterans [14].

During consultations held in Weeks 1, 4, and 8, participants will create their individualized Active Choices program, with the support of a consultant, for the proceeding 4 weeks; consistent with our sample size calculations and main outcome measure, focus will be on encouraging weekly activities that elicit self-managed moderate intensity PA equivalent to the recommended guidelines of 150 minutes/week [12], although lighter and more vigorous intensity activities will also be options clients can include in their program where appropriate. This will involve identifying PA preferences and linking participants to preferred activities in their local community, setting PA goals, developing a PA action plan, identifying barriers to PA, and problem-solving solutions to overcome these. Participants will also be actively linked to other participants in their area to facilitate the formation of PA social groups. In addition to these program elements, education about PA will be delivered to participants in Week 1, and a review of previously set PA goals will be conducted in Weeks 4 and 8. At the final consultation in Week 12, participants will review their progress throughout the program and develop a plan for continuing to self-manage PA.

Consultations will be guided by a resource booklet that contains educational and behavioral support materials that map to the behavior change strategies. This booklet will also be used by participants throughout the 12-week program as an educational resource and to self-monitor their PA. A sample of this booklet is provided in Multimedia Appendix 1. SMS reminders will be sent to participants the day before their PA choices in Weeks 2, 5, and 9 as a behavioral prompt.

#### Comparison Program

As Figure 1 describes, participants attending EP or physiotherapy practices allocated to the comparison group will receive a 12-week program that is more self-directed, has fewer consultation sessions, and incorporates fewer behavioral support strategies than the Active Choices program (ie, education, self-monitoring, and action planning only). The comparison program consists of two 1-hour face-to-face consultations (held in Weeks 1 and 12) that participants will complete individually with an Active Choices consultant. During the consultation in Week 1, participants will receive education about PA and identify their PA preferences. They will also be given materials to help them identify local opportunities for their PA choices and to develop their PA action plan independently at home. At the consultation in Week 12, participants will reflect on their progress during the past 12 weeks and identify their PA action plan for continuing to self-manage PA. Consultations will be guided by a resource booklet, and participants will use this booklet to self-monitor their PA across the 12-week program.

#### Concomitant Care

Involvement in the research will not replace existing treatment plans but seek to “value-add” scaffolding for behavioral support that can benefit clients and the PA specialist. Therefore, participants in practices allocated to both programs will continue to have the option of accessing allied health treatment from their EP or physiotherapist during the 24-week study period.

### Outcome Measures

#### Overview

The primary outcome of self-managed moderate intensity PA and secondary outcomes of psychological well-being and social connectedness will be assessed at baseline, end-intervention (Week 12), and follow-up (Week 24; see Figure 1). In addition, physical measurements of height and weight and a lifestyle survey assessing quality of life and health behaviors (nutrition, smoking, and alcohol use) will be completed at these time points. Standard demographic items assessing age, gender, education, employment, and household status will be administered at baseline.

Measures will be administered by trained researchers and standardized with calibration of devices and equipment prior to measurement sessions. An Aus $50 (US $38.71) grocery voucher will be offered to incentivize participants to complete follow-up measures at 24 weeks.

#### Primary Outcome Measure

The primary outcome measure of Active Choices is moderate intensity self-managed PA (equivalent to recommended guidelines of 150 minutes/week [12]) assessed by accelerometer devices. Accelerometers are now widely recognized as an affordable, practical, and highly accurate means of assessing PA [15]. We will use a triaxial accelerometer (wGT3X+, ActiGraph, Pensacola, FL) to assess PA using the same protocol at the 3 measurement time points. Participants will wear the device on their nondominant wrist for 7 consecutive days (24 hours) and keep a diary to record times when they attended treatment with their EP or physiotherapist, when the device was removed, and sleep hours. Upon return, the raw data from the device will be downloaded and processed. The 24-hour wear-time protocol will record raw acceleration and will be used to quantify overall time in moderate PA (inclusive of land and water-based activities), as well as time spent in light and vigorous intensity activities, sedentary behavior, and sleep. Moderate intensity self-managed PA will be calculated as the difference between total moderate intensity PA minus supervised PA performed during treatment (recorded in the diary).

#### Secondary Outcome Measures

Psychological well-being will be assessed using the Satisfaction with Life Scale [16]*.* This is a widely used, validated instrument that is comprised of 5 items. Responses to each item are made using a 7-point Likert scale (1 = strongly disagree; 7 = strongly agree).

Social connectedness will be assessed using the New Group Membership Scale (NGMS), Social Identity Mapping, and Three-Item Loneliness Scale. The NGMS is comprised of 4 items that assess the extent to which people have joined new social groups and has strong internal reliability [17]. Responses are rated using a 7-point Likert scale (1 = strongly disagree; 7 = strongly agree). The NGMS will be used to determine whether engagement in PA provides a platform to extend people’s social networks to impact on inclusivity. Social Identity Mapping is a validated online tool of social connectedness assessing the multidimensional and connected nature of people’s social group networks (eg, family, work, arts-based, sports) and associated social identities (eg, as a veteran, member of a cycling club, or yoga class). Its elements—that comprise group importance, support, positivity, representativeness, and compatibility—are recognized predictors of a range of health and well-being outcomes. This project will use the latest online version validated in 5 studies [18]. The Three-Item Loneliness Scale is a validated and reliable measure of loneliness and social isolation. It is comprised of 3 items, with responses made using a 3-point scale (1 = hardly ever; 2 = some of the time; 3 = often) [19].

### Process and Qualitative Measures

Self-report data logged by participants in the resource booklet will capture the frequency, quantity, and types of PA participants in both groups engaged with during the 12-week intervention. We will also conduct interviews with participants in both groups at end-intervention and follow-up to explore the extent to which program experiences promoted social connectedness and veteran social networks, aspects of the program that clients found beneficial and enjoyed, and aspects that could be further developed to improve program efficacy.

### Health Service Data

In addition to the measures that will be administered through the trial, we will also seek DVA data custodian approval to access participants’ deidentified health service data. We will use these data to assess treatment history prior to recruitment and health service utilization and costs to the DVA for providing these services during the intervention and follow-up period. This will enable us to determine the cost consequences of the program.

### Data Analyses

Baseline descriptive statistics will be used to summarize the demographic, physical, and lifestyle behavior characteristics of participants, and data will be checked for parametric assumptions. Intention-to-treat analyses will be conducted using linear mixed effects models. This approach will be used to account for the repeated outcome measures over time and the clustered design of the study and will provide estimates on the within-group and between-group changes in accelerometer-measured moderate intensity PA, psychological well-being, and social connectedness. Sensitivity analyses will be conducted to assess the robustness of findings, and for exploratory purposes, subgroup analyses will be performed by gender, age group, and treatment history. The results of all comparative analyses will be presented with 95% confidence intervals, and statistical significance for main effects will be assessed at the 5% level. Statistical analyses will be conducted using Stata (v15; StataCorp LLC, College Station, TX).

To analyze cost consequences, we will determine the costs of Active Choices and of normal treatment alone, inclusive of both the direct costs of implementing the intervention and the implied cost to the DVA (such as the higher or lower utilization of EP or physiotherapist services relative to group allocation). Difference-in-difference analysis will be used to determine whether Active Choices contributes to cost savings, due to a possible reduction in service utilization.

Consistent with recognized guidelines for qualitative data analyses [20], members of our research team will thematically analyze and independently review interview data and discuss the range of responses to agree on key themes. We will use this qualitative approach to triangulate our findings from the statistical analyses (eg, the impacts of the program on self-managed PA) and determine the factors that encouraged or discouraged engagement with the Active Choices program.

### Accelerometer Data Processing

Raw data will be processed in R using the most up-to-date GGIR package, a widely used open-source code [21]. This will involve a calibration to local gravity [22], adjustment for nonwear time, and a filter for abnormally high values. Nonwear time will be defined as periods of at least 60 consecutive minutes with low acceleration variability [23]. The vector magnitude of the 3 axes will be used to calculate activity-related acceleration using Euclidian Norm minus 1g [ENMO=√(x2+y2+z2)-1]. For segments with invalid data, the average of similar time-of-day data points from other days of measurement in the same individual will be imputed. Data will be included if wear time is at least 600 minutes/day on 4 or more days. Data will be used to quantify overall PA expressed as acceleration in milligravity units (mg), as well as time spent in activities at different intensities using the intensity thresholds proposed by Hildebrand et al [24]: light intensity, acceleration 30-100 mg; moderate intensity, acceleration 100-400 mg; vigorous intensity, acceleration higher than 400 mg.

### Ethics Approval

The study has received approval from The Departments of Defence and Veterans’ Affairs Human Research Ethics Committee (DDVAHREC/OUT/2019/BN11979933; December 13, 2019) and The University of Queensland Human Research Ethics Committee (#2020000034; January 31, 2020).

## Results

The study was funded by the DVA in April 2019. Liaison with EP and physiotherapy practices began in February 2020. However, the study was suspended on March 20, 2020 due to the COVID-19 pandemic and restrictions to face-to-face research activities. Our aim is to commence participant recruitment when it is feasible and safe to do so. In our project timeline, the target duration for participant recruitment is 3 months, with study implementation planned to run for 9 months. We expect that results will be available 6 months after data collection is completed.

## Discussion

The purpose of this study is to test Active Choices, a stepped-down behavioral support program to help DVA clients (ADF veterans and their dependents) self-manage PA as they transition from treatment by an EP or physiotherapist. Findings from the systematic review recently conducted by our research group suggest that such programs have the potential to promote short-term PA changes in US military service veterans with high-risk comorbidities (eg, diabetes, posttraumatic stress disorder, musculoskeletal disorders). Among those selected studies that compared a stepped-down intervention to a “usual care” group (n=14), 79% of studies (11/14) observed a positive between-group intervention effect, with the mean magnitude of change being 53 minutes/week of self-reported moderate intensity PA [14].

While this evidence is promising, our review identified no studies with ADF veterans. Similarities may well exist with US military service veterans; however, there is no direct evidence of generalizability to the Australian context or indeed outside of the United States to military service veterans in other countries. The proposed study will therefore contribute important evidence to an identified research need, and the findings provide valuable pilot data to inform larger effectiveness trials at the national level. Beyond this, our study will also address other limitations in the veteran evidence base. These include the use of self-report rather than objective measures of PA change and importantly, lack of data concerning the extent to which self-managed PA programs can benefit the psychological well-being and social connectedness of veterans or their dependents.

## Appendix

Multimedia Appendix 1 Sample from Active Choices Resource Booklet.

## Abbreviations

ADF: Australian Defence Force
DVA: Department of Veterans’ Affairs
EP: exercise physiologist
NGMS: New Group Membership Scale
PA: physical activity

## References

[1] (2018). Global action plan on physical activity 2018-2030: More active people for a healthier world. World Health Organization.

[2] Bauman A, Merom D, Bull FC, Buchner DM, Fiatarone Singh MA (2016). Updating the Evidence for Physical Activity: Summative Reviews of the Epidemiological Evidence, Prevalence, and Interventions to Promote. Gerontologist.

[3] Craike M, Britt H, Parker A, Harrison C (2019). General practitioner referrals to exercise physiologists during routine practice: A prospective study. J Sci Med Sport.

[4] (2018). Treatment Population Statistics: Quarterly Report - June 2018. Australian Government Department of Veterans' Affairs.

[5] Buckley BJR, Thijssen DHJ, Murphy RC, Graves LEF, Whyte G, Gillison FB, Crone D, Wilson PM, Watson PM (2018). Making a move in exercise referral: co-development of a physical activity referral scheme. J Public Health (Oxf).

[6] (2012). Step-down care. Segen's Medical Dictionary.

[7] Haibach JP, Haibach MA, Hall KS, Masheb RM, Little MA, Shepardson RL, Dobmeyer AC, Funderburk JS, Hunter CL, Dundon M, Hausmann LRM, Trynosky SK, Goodrich DE, Kilbourne AM, Knight SJ, Talcott GW, Goldstein MG (2017). Military and veteran health behavior research and practice: challenges and opportunities. J Behav Med.

[8] Kanamori S, Takamiya T, Inoue S, Kai Y, Kawachi I, Kondo K (2016). Exercising alone versus with others and associations with subjective health status in older Japanese: The JAGES Cohort Study. Sci Rep.

[9] Hatch SL, Harvey SB, Dandeker C, Burdett H, Greenberg N, Fear NT, Wessely S (2013). Life in and after the Armed Forces: social networks and mental health in the UK military. Sociol Health Illn.

[10] Schrempft S, Jackowska M, Hamer M, Steptoe A (2019). Associations between social isolation, loneliness, and objective physical activity in older men and women. BMC Public Health.

[11] Burton NW, Haynes M, Wilson LM, Giles-Corti B, Oldenburg BF, Brown WJ, Giskes K, Turrell G (2009). HABITAT: A longitudinal multilevel study of physical activity change in mid-aged adults. BMC Public Health.

[12] (2019). Australia's Physical Activity and Sedentary Behaviour Guidelines and the Australian 24-Hour Movement Guidelines. Australian Government Department of Health.

[13] Michie S, van Stralen MM, West R (2011). The behaviour change wheel: a new method for characterising and designing behaviour change interventions. Implement Sci.

[14] Gilson ND, Papinczak ZE, Mielke GI, Haslam C, McKenna J, Brown WJ (2019). ‘Stepped-down’ Intervention Programs to Promote Self-Managed Physical Activity in Service Veterans and their Dependants: Technical Report. Department of Veterans’ Affairs.

[15] Esliger DW, Tremblay MS (2007). Physical activity and inactivity profiling: the next generation. Can J Public Health.

[16] Diener E, Emmons RA, Larsen RJ, Griffin S (1985). The Satisfaction With Life Scale. J Pers Assess.

[17] Haslam C, Holme A, Haslam SA, Iyer A, Jetten J, Williams WH (2008). Maintaining group memberships: social identity continuity predicts well-being after stroke. Neuropsychol Rehabil.

[18] Cruwys T, Steffens NK, Haslam SA, Haslam C, Jetten J, Dingle GA (2016). Social Identity Mapping: A procedure for visual representation and assessment of subjective multiple group memberships. Br J Soc Psychol.

[19] Hughes ME, Waite LJ, Hawkley LC, Cacioppo JT (2004). A Short Scale for Measuring Loneliness in Large Surveys: Results From Two Population-Based Studies. Res Aging.

[20] Tong A, Sainsbury P, Craig J (2007). Consolidated criteria for reporting qualitative research (COREQ): a 32-item checklist for interviews and focus groups. Int J Qual Health Care.

[21] Migueles JH, Rowlands AV, Huber F, Sabia S, van Hees VT (2019). GGIR: A Research Community-Drive Open Source R Package for Generating Physical Activity and Sleep Outcomes From Multi-Day Raw Accelerometer Data. Journal for the Measurement of Physical Behaviour.

[22] van Hees VT, Fang Z, Langford J, Assah F, Mohammad A, da Silva ICM, Trenell MI, White T, Wareham NJ, Brage S (2014). Autocalibration of accelerometer data for free-living physical activity assessment using local gravity and temperature: an evaluation on four continents. J Appl Physiol (1985).

[23] van Hees VT, Gorzelniak L, Dean León EC, Eder M, Pias M, Taherian S, Ekelund U, Renström F, Franks PW, Horsch A, Brage S (2013). Separating movement and gravity components in an acceleration signal and implications for the assessment of human daily physical activity. PLoS One.

[24] Hildebrand M, Van Hees VT, Hansen BH, Ekelund U (2014). Age group comparability of raw accelerometer output from wrist- and hip-worn monitors. Med Sci Sports Exerc.

