# Supplementary material for: Effects of the Active Choices Program on Self-Managed Physical Activity and Social Connectedness in Australian Defence Force Veterans: Protocol for a Cluster-Randomized Trial
Source: JMIR Res Protoc. 2021 Feb 24;10(2):e21911. doi: 10.2196/21911 (PMC7946582; doi:10.2196/21911)
Supplement: Multimedia Appendix 1 [file resprot_v10i2e21911_app1.pdf]

THE UNIVERSITY OF QUEENSLAND
AUSTRALIA
CREATE CHANGE

Active Choices

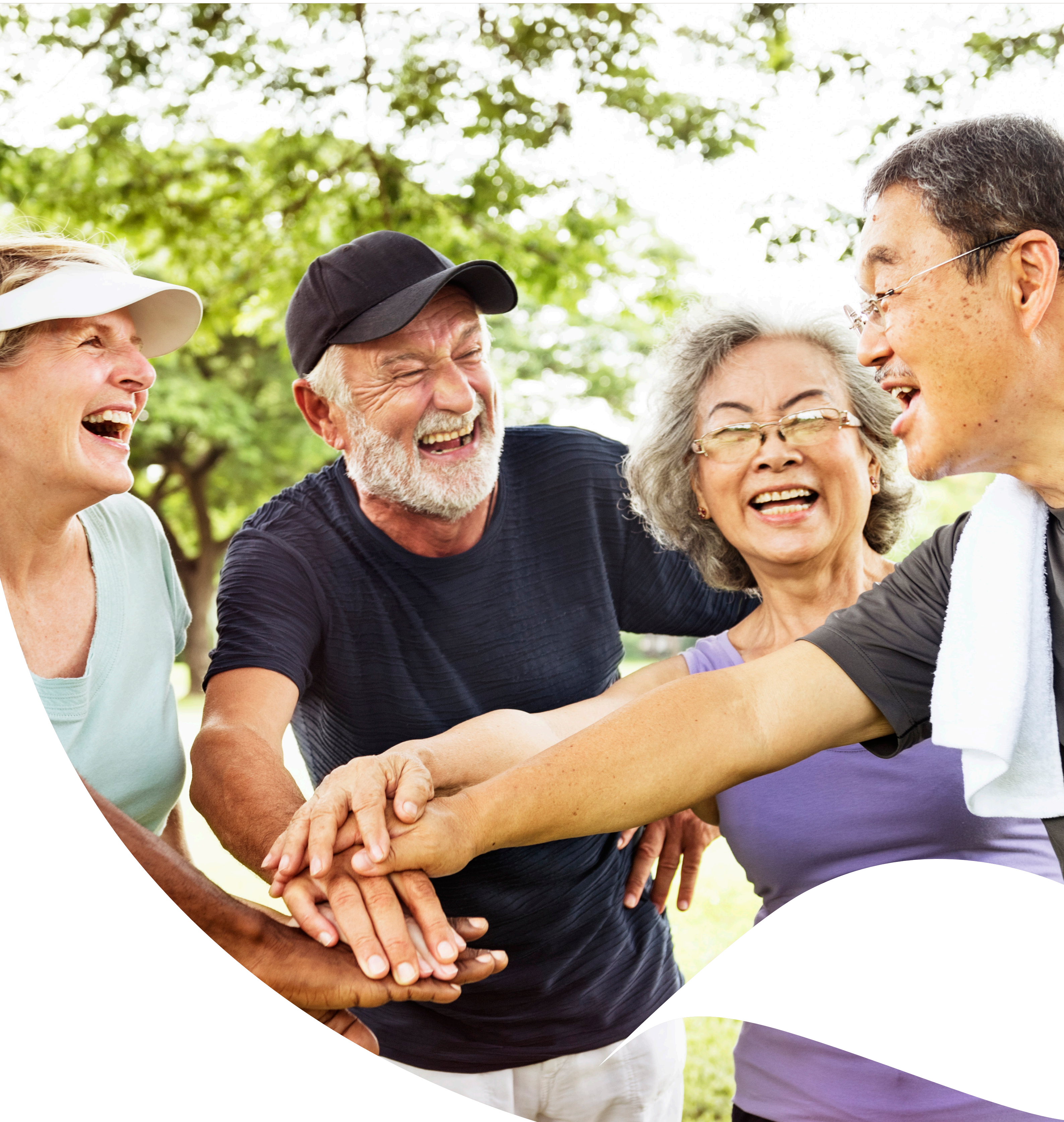

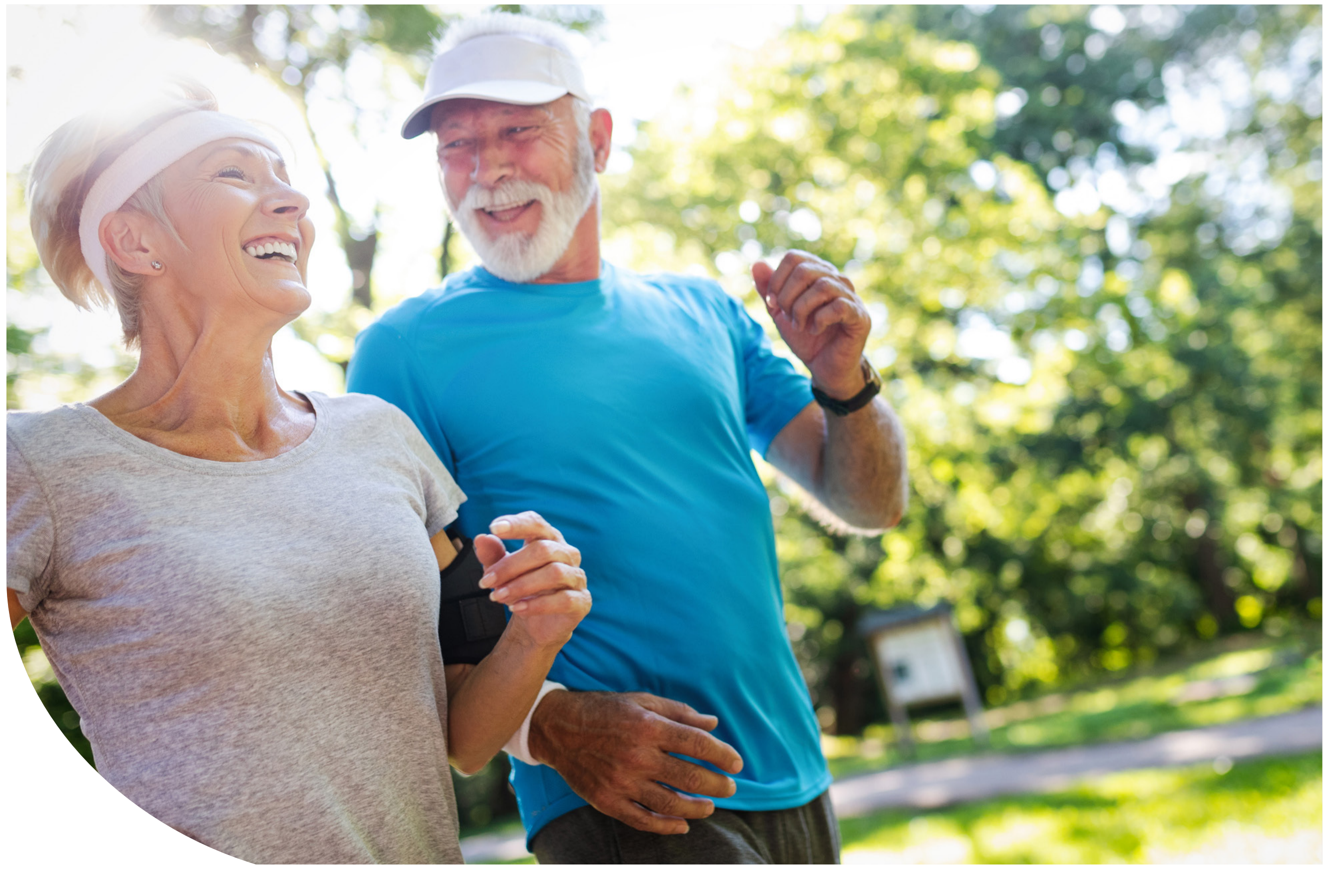

About *Active Choices*

Active Choices is a 12-week program that helps you be more physically active and socially connected within your local community.

The program aims to link you to fun, social, local group activities of your choosing, and provide you with support strategies to self-manage your physical activity during and after the program ends.

About this booklet

This resource booklet will support you during the *Active Choices* program. It contains educational materials and helpful tips to become and remain physically active. It also contains materials and tools for choosing and managing the physical activities you want to try.

Particular sections of the booklet we'll discuss at our initial consultation, when we check-in with you by telephone in weeks 4 and 8, and when you return for your final consultation meeting in week 12. Other sections of the booklet, like the physical activity log, will be completed by you independently at home.

Regular reference to the information and materials in this booklet throughout the program will support you in achieving your physical activity goals.

If you have any questions about the contents of this booklet or the *Active Choices* program, be sure to ask us.

Program Structure

Week 0: Start measures of your physical activity and health

↓

Week 1 to 12: *Active Choices* Program

↓

Week 1: Initial Consultation – Getting Started
- Develop your personal *Active Choices* program for weeks 1 to 4

↓

Week 4: Telephone Check-In
- Review your progress during weeks 1 to 4
- Develop your personal *Active Choices* program for weeks 5 to 8

↓

Week 8: Telephone Check-In
- Review your progress during weeks 5 to 8
- Develop your personal *Active Choices* program for weeks 9 to 12

↓

Week 12: Final Consultation - Moving Forward
- Review your progress during weeks 9 to 12
- Develop your plan for self-managing physical activity into the future

↓

Week 13 to 24: Self-Manage Physical Activity

↓

Week 24: Final measures of your physical activity and health

Active Choices

Contents

About *Active Choices*	1
Program Structure	2
Contents Page	3
Making sure your activities are safe	5
Why is physical activity important?	6
How physically active are you?	7
What are the different types of physical activity?	8
What types of physical activity would you like to do?	9
Your local opportunities for physical activity	10
Your physical activity goals | Week 1 to 4	11
Your physical activity plan | Week 1 to 4	12
Tips for staying physically active	13
Physical activity logbook | Week 1 to 4	15
Week 4 Telephone Check-In	17
Physical activity logbook | Week 5 to 8	21
Week 8 Telephone Check-In	23
Physical activity logbook | Week 9 to 12	27
Week 12 Consultation	29

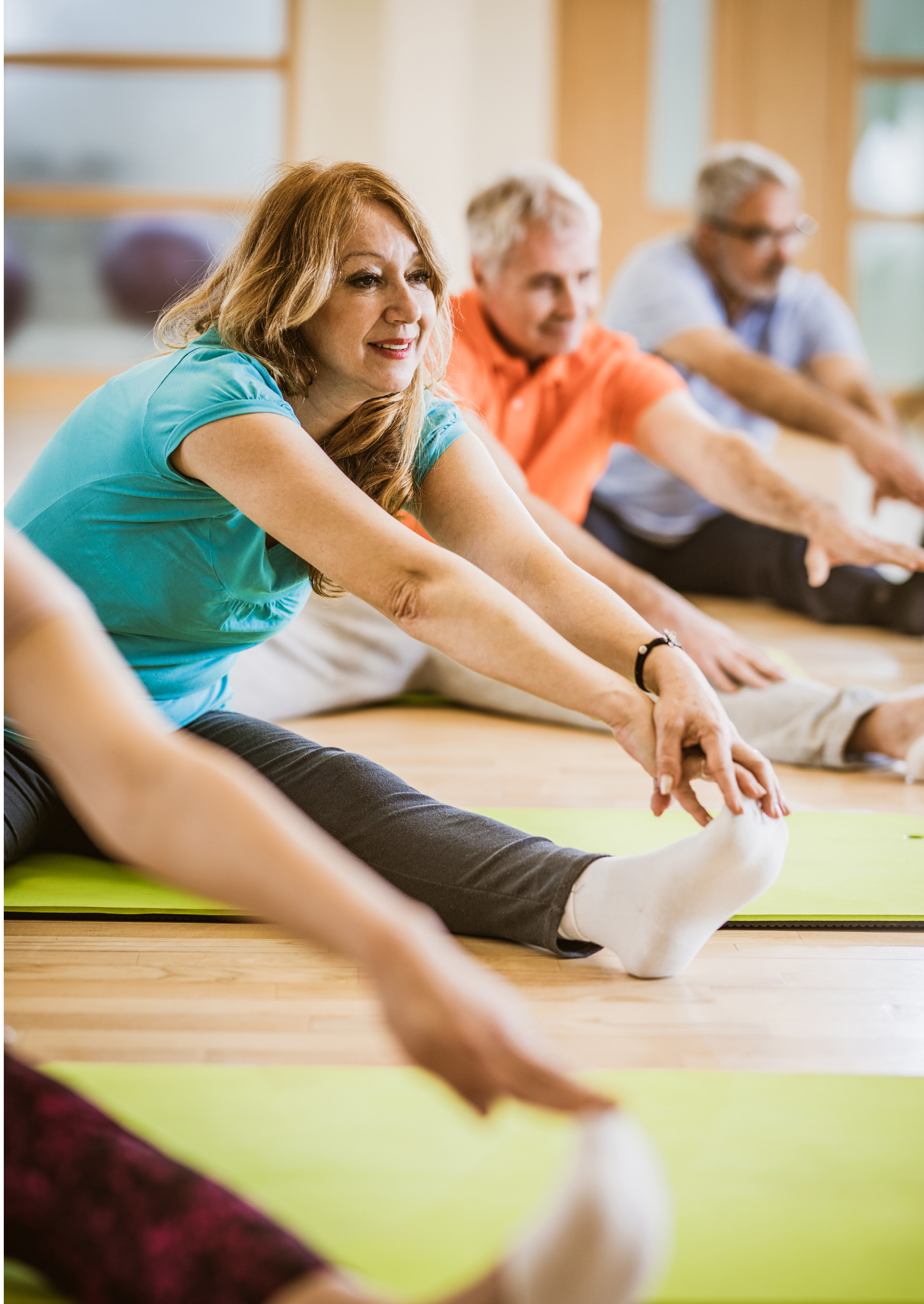

Physical activity logbook | Week 1 to 4

During the course of the *Active Choices* program, we would like you to keep a record of the physical activity you do each day. Through self-monitoring your physical activity, you will be able to keep track of your progress and assess whether you are meeting your goals.

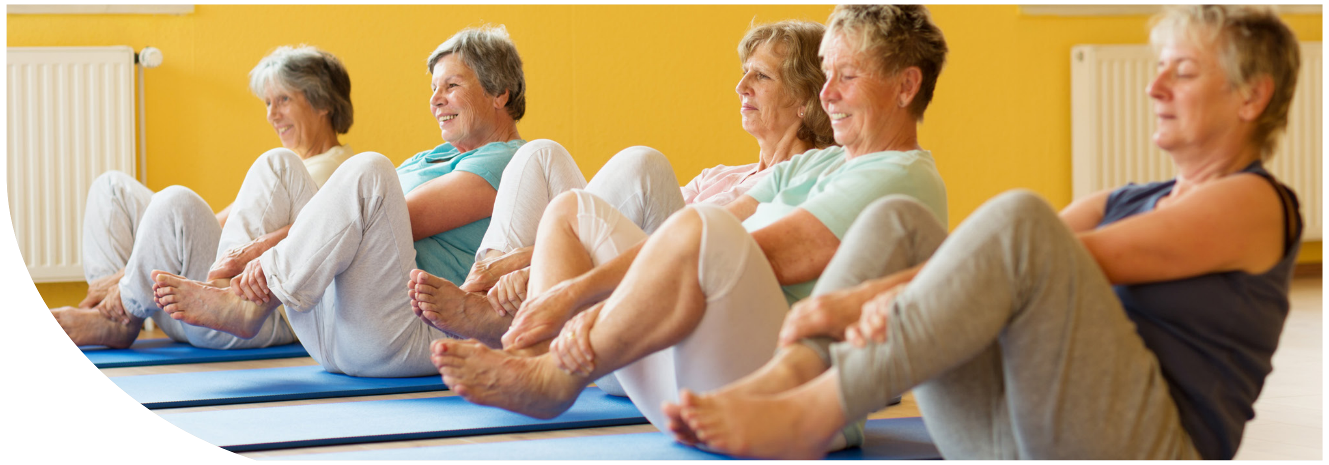

Using the logbook provided, please record the following details about the physical activity you complete during weeks 1 to 4 of the program:

- The **type of activity** you did (e.g., Pilates class) – write the name of the activity in the 'activity' column.
- The **amount of time** you did the activity for (e.g., 60 minutes) – write the number of minutes in the 'day of the week' box.

An example of how to fill in the logbook is provided below.

Activity	Week 1							Week 2							Week 3							Week 4						
	Sun	Mon	Tue	Wed	Thur	Fri	Sat	Sun	Mon	Tue	Wed	Thur	Fri	Sat	Sun	Mon	Tue	Wed	Thur	Fri	Sat	Sun	Mon	Tue	Wed	Thur	Fri	Sat
Walking group	60							60							60							60						
Pilates class		60							60							60						60						

Week 4: Telephone Check-In

We will check in with you in four weeks time to see how you are going with your *Active Choices* program. Using your self-monitoring log, we will work with you to review your goal progress. We'll also see if you'd like to change or continue with your *Active Choices* and help you set your goals for weeks 5 to 8.

Let's now agree on a time for your Week 4 Telephone Check-In:

**Date:**	**Time:**
